# Ammonium diphosphitoindate(III)

**DOI:** 10.1107/S160053681300771X

**Published:** 2013-03-28

**Authors:** Farida Hamchaoui, Houria Rebbah, Eric Le Fur

**Affiliations:** a Laboratoire Sciences des Matériaux, Faculté de Chimie, Université des Sciences et de la Technologie Houari Boumediene, BP 32 El-Alia, 16111 Bab-Ezzouar Alger, Algeria; b Ecole Nationale Supérieure de Chimie de Rennes, CNRS, UMR 6226, Avenue du Général Leclerc CS 50837, 35708 Rennes Cedex 7, France

## Abstract

The crystal structure of the title compound, NH_4_[In(HPO_3_)_2_], is built up from In^III^ cations (site symmetry 3*m*.) adopting an octa­hedral environment and two different phosphite anions (each with site symmetry 3*m*.) exhibiting a triangular–pyramidal geometry. Each InO_6_ octa­hedron shares its six apices with hydrogen phosphite groups. Reciprocally, each HPO_3_ group shares all its O atoms with three different metal cations, leading to [In(HPO_3_)_2_]^−^ layers which propagate in the *ab* plane. The ammonium cation likewise has site symmetry 3*m*.. In the structure, the cations are located between the [In(HPO_3_)_2_]^−^ layers of the host framework. The sheets are held together by hydrogen bonds formed between the NH_4_^+^ cations and the O atoms of the framework.

## Related literature

For general background, see: Natarajan & Mandal (2008 ▶); Marcos *et al.* (1993 ▶). For related structures, see: Li *et al.* (2013 ▶); Hamchaoui *et al.* (2013 ▶); Giester (2000 ▶); Graeber & Rosen­zweig (1971 ▶). For potential applications of open-framework transition metal phosphates, see: Cheetham *et al.* (1999 ▶). For the synthesis of the first organically templated vanadium phosphite with an open framework, see: Bonavia *et al.* (1995 ▶). Structures of purely inorganic phosphite compounds have been evidenced with magnetic and non-magnetic cations (Marcos *et al.*, 1993 ▶; Morris *et al.*, 1994 ▶; Orive *et al.*, 2011 ▶) while closely related structures can be obtained by replacing organic cations by inorganic ones as observed in the *A_x_*Mn_3_(HPO_3_)_4_ system [*A* = en (Fernández *et al.*, 2000 ▶); *A* = K (Hamchaoui *et al.*, 2009 ▶)].

## Experimental

### 

#### Crystal data

NH_4_[In(HPO_3_)_2_]
*M* *_r_* = 292.82
Hexagonal, 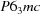

*a* = 5.4705 (1) Å
*c* = 13.0895 (4) Å
*V* = 339.24 (1) Å^3^
*Z* = 2
Mo *K*α radiation
μ = 3.93 mm^−1^
*T* = 293 K
0.10 × 0.05 × 0.02 mm

#### Data collection

Nonius KappaCCD diffractometer
Absorption correction: multi-scan (*SADABS*; Sheldrick, 2002) ▶ *T*_min_ = 0.66, *T*_max_ = 0.92
7774 measured reflections
962 independent reflections
912 reflections with *I* > 2σ(*I*)
*R*_int_ = 0.024

#### Refinement

*R*[*F* ^2^ > 2σ(*F* ^2^)] = 0.017
*wR*(*F* ^2^) = 0.041
*S* = 1.26
962 reflections
30 parameters
5 restraints
H atoms treated by a mixture of independent and constrained refinement
Δρ_max_ = 0.51 e Å^−3^
Δρ_min_ = −1.39 e Å^−3^
Absolute structure: Flack (1983 ▶), 459 Friedel pairs
Flack parameter: −0.01 (2)

### 

Data collection: *COLLECT* (Nonius, 1998 ▶); cell refinement: *DIRAX/LSQ* (Duisenberg, 1992 ▶); data reduction: *EVALCCD* (Duisenberg, 1998 ▶); program(s) used to solve structure: *SIR97* (Altomare *et al.*, 1999 ▶); program(s) used to refine structure: *SHELXL97* (Sheldrick, 2008 ▶); molecular graphics: *DIAMOND* (Brandenburg, 2005 ▶); software used to prepare material for publication: *WinGX* (Farrugia, 2012 ▶).

## Supplementary Material

Crystal structure: contains datablock(s) global, I. DOI: 10.1107/S160053681300771X/ru2050sup1.cif

Structure factors: contains datablock(s) I. DOI: 10.1107/S160053681300771X/ru2050Isup2.hkl

Additional supplementary materials:  crystallographic information; 3D view; checkCIF report

## Figures and Tables

**Table 1:** Hydrogen-bond geometry (Å, °)

*D*—H⋯*A*	*D*—H	H⋯*A*	*D*⋯*A*	*D*—H⋯*A*
N1—H*N*1⋯O1	0.86 (2)	2.38 (2)	3.066 (2)	138 (2)
N1—H*N*1⋯O1^i^	0.86 (2)	2.38 (2)	3.066 (2)	138 (2)
N1—H*N*2⋯O2^ii^	0.89 (1)	2.41 (1)	3.109 (4)	135 (1)
N1—H*N*2⋯O2^iii^	0.89 (1)	2.41 (1)	3.109 (4)	135 (1)
N1—H*N*2⋯O2^iv^	0.89 (1)	2.41 (1)	3.109 (4)	135 (1)
N1—H*N*2⋯O2^v^	0.89 (1)	2.41 (1)	3.109 (4)	135 (1)

Symmetry codes: (i) 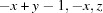; (ii) 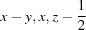; (iii) 
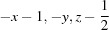
; (iv) 
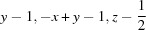
; (v) 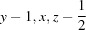.

**Fig. 1.**
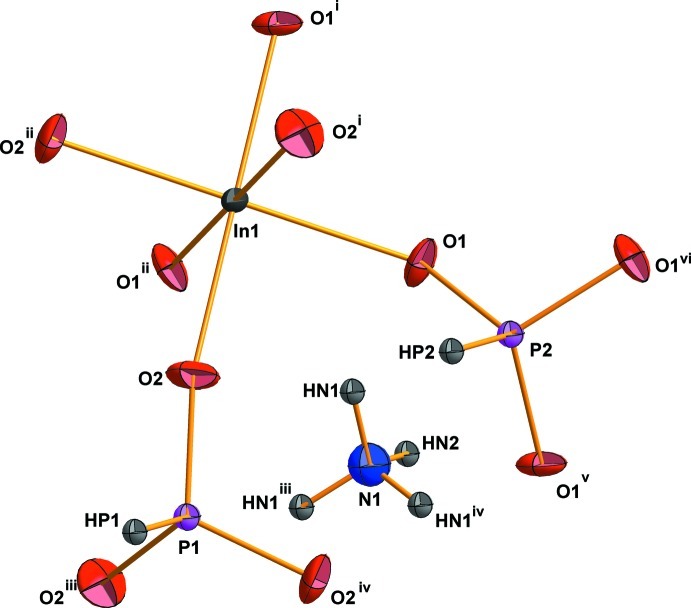
The asymmetric unit and symmetry-related atoms of NH4[In(HPO3)2], shown with 50% probability displacement ellipsoids. [symmetry codes: (i) -y, x-y + 1, z; (ii) -x + y - 1, -x, z; (iii) -y - 1, x-y, z; (iv) -x + y - 1, -x - 1, z; (v) -y, x-y, z; (vi) -x + y, -x, z].

**Fig. 2.**
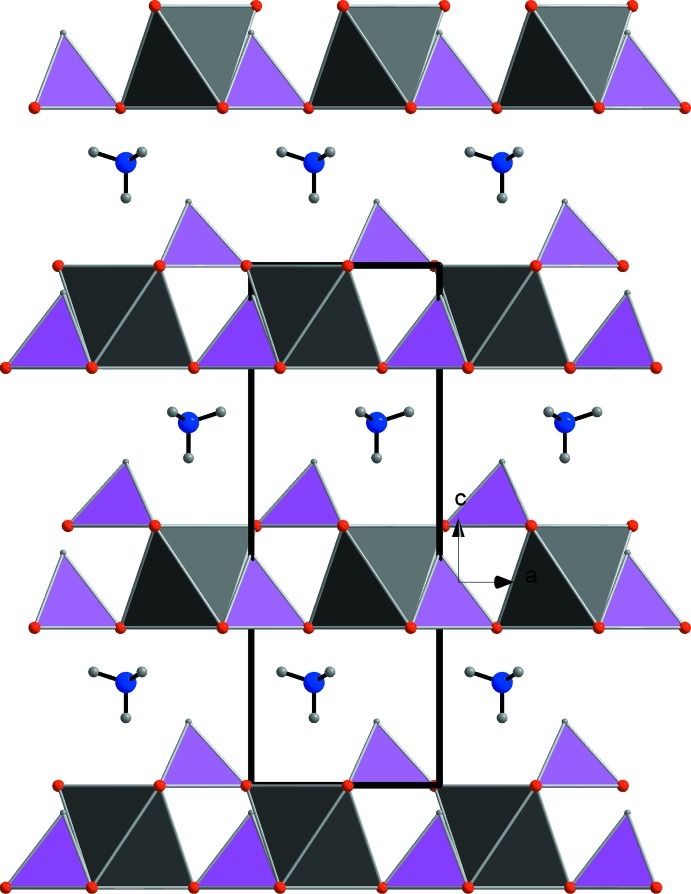
Projection along the [010] direction, showing the two-dimensional framework in NH4[In(HPO3)2].

**Fig. 3.**
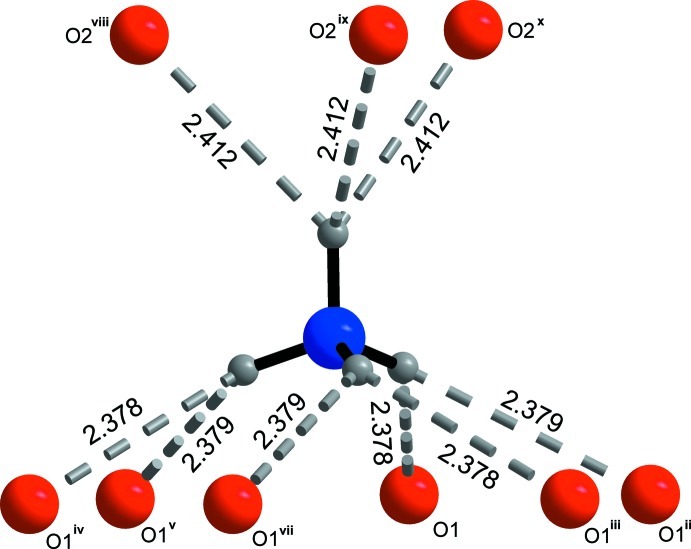
H-bonding arrangement between the ammonium cations and the host framework [symmetry codes: (ii) -x + y - 1, -x, z; (iii) -y - 1, x-y, z; (iv) -x + y - 1, -x - 1, z; (v) -y, x-y, z; (vii) x - 1, y - 1, z; (viii) x-y, x, -0.5 + z, (ix) y - 1, -x + y - 1, -0.5 + z; (x) -x - 1, -y, -0.5 + z].

## supplementary crystallographic information

### Comment

After the discovery of microporous aluminophosphates, considerable efforts have been directed towards the synthesis of new open-framework transition metal phosphates because of their potential applications (Cheetham *et al.*, 1999). The replacement of phosphate by phosphite in transition metal phosphates has recently attracted effort, notably since the synthesis of the first organically templated vanadium phosphite with an open framework (Bonavia *et al.*, 1995). Consequently the literature is currently dominated by reports of organically template phosphite frame- works (Natarajan & Mandal, 2008). Purely inorganic phosphite structures have also been evidenced with magnetic and non-magnetic cations (Marcos *et al.*, 1993, Morris *et al.*, 1994, Orive *et al.*, 2011) while, interestingly, closely related structures can be obtained by replacing organic cations by inorganic ones as observed in the A*_x_*Mn_3_(HPO_3_)_4_ system. (A = en: Fernández *et al.*, 2000, A = K: Hamchaoui *et al.*, 2009).

The structure of the title compound is built up from In(HPO_3_)_2_ layers separated by NH_4_ cations. It is isostructural to (H_3_O)In(HPO_3_)_2_ (Li *et al.*, 2013) and to the A[*M*(HPO_3_)_2_] family (A = K, Rb, NH_4_ and *M* = V, Fe) (Hamchaoui *et al.*, 2013). The structural model is also related to the yavapaiite aluns type (Graeber & Rosenzweig, 1971) and the mixed selenite-selenate [(RbFe(SeO_4_)(SeO_3_)](Giester, 2000). As shown in Fig. 1, the asymmetric unit contains one crystallographic independant In^III^ cation and two ones for both phosphorus and oxygen atoms. Six oxygen atoms define an octahedral geometry around the metallic center while three oxygen atoms and one hydrogen atom define the triangular pyramidal environment of the phosphorus atom. The quaternary ammonium ions are displayed between the [*M*(HPO_3_)_2_]^-^ layers of the host framework (Fig. 2). They exhibit N—H bond distances in the range usually found for this cation and the angles are similar to those expected for *sp^3^* hybridization. Thus for 1 the sheets are held together by hydrogen bonds formed between and the oxygen atoms of the framework. This H-bonding arrangement is illustrated in Fig. 3. It shows that the ammonium ion is firmly fixed in the structure by means of nine N—H···O hydrogen bonds, which prevent free ammonium-ion rotation at 298 K. The ammonium cations are located at the center of the six-ring windows of the upper layer.

### Experimental

The title compound was prepared under mild hydrothermal conditions and autogenous pressure. The starting reagents were InCl_3_ (Sigma-Aldrich, 98%), H_3_PO_3_ (Aldrich, 99%), (NH_4_)_2_CO_3_ (Fluka, 30–33% of NH_3_) and deionized water in a 2:15:4:280 molar ratio. The mixture was placed in a 23 ml Teflon-lined steel autoclave, heated at 453 K for 72 h and followed by slow cooling to room temperature. Well formed colorless crystals were recovered by vacuum filtration, washed with deionized water and dried in a desiccator.

### Refinement

Part of the H atoms was localized from a difference Fourier map (HP2 and HN1) others were placed in calculated position according to geometrical constraints. Hydrogen atom positions of the ammonium cation were refined with their N—H and H—H distances restrained to one common refined value (0.87 Å and 1.33 Å respectively)

### Crystal data

**Table:**

NH_4_[In(HPO_3_)_2_]	*D*_x_ = 2.867 Mg m^−^^3^
*M**_r_* = 292.82	Mo *K*α radiation, λ = 0.71073 Å
Hexagonal, *P*6_3_*m**c*	Cell parameters from 1590 reflections
*a* = 5.4705 (1) Å	θ = 2.9–42.1°
*c* = 13.0895 (4) Å	µ = 3.93 mm^−^^1^
*V* = 339.24 (1) Å^3^	*T* = 293 K
*Z* = 2	Block, colourless
*F*(000) = 280	0.1 × 0.05 × 0.02 mm

### Data collection

**Table:**

Nonius KappaCCD diffractometer	962 independent reflections
Radiation source: fine-focus sealed tube	912 reflections with *I* > 2σ(*I*)
Graphite monochromator	*R*_int_ = 0.024
CCD rotation images, thick slices scans	θ_max_ = 42.0°, θ_min_ = 4.3°
Absorption correction: multi-scan (*SADABS*; Sheldrick, 2002)	*h* = −10→9
*T*_min_ = 0.66, *T*_max_ = 0.92	*k* = −10→9
7774 measured reflections	*l* = −24→24

### Refinement

**Table:**

Refinement on *F*^2^	Hydrogen site location: inferred from neighbouring sites
Least-squares matrix: full	H atoms treated by a mixture of independent and constrained refinement
*R*[*F*^2^ > 2σ(*F*^2^)] = 0.017	*w* = 1/[σ^2^(*F*_o_^2^) + (0.0183*P*)^2^ + 0.0737*P*] where *P* = (*F*_o_^2^ + 2*F*_c_^2^)/3
*wR*(*F*^2^) = 0.041	(Δ/σ)_max_ = 0.003
*S* = 1.26	Δρ_max_ = 0.51 e Å^−^^3^
962 reflections	Δρ_min_ = −1.39 e Å^−^^3^
30 parameters	Extinction correction: *SHELXL97* (Sheldrick, 2008), Fc^*^=kFc[1+0.001xFc^2^λ^3^/sin(2θ)]^-1/4^
5 restraints	Extinction coefficient: 0.092 (4)
Primary atom site location: structure-invariant direct methods	Absolute structure: Flack (1983), 459 Friedel pairs
Secondary atom site location: difference Fourier map	Flack parameter: −0.01 (2)

### Special details

**Table:**

Geometry. All e.s.d.'s (except the e.s.d. in the dihedral angle between two l.s. planes) are estimated using the full covariance matrix. The cell e.s.d.'s are taken into account individually in the estimation of e.s.d.'s in distances, angles and torsion angles; correlations between e.s.d.'s in cell parameters are only used when they are defined by crystal symmetry. An approximate (isotropic) treatment of cell e.s.d.'s is used for estimating e.s.d.'s involving l.s. planes.
Refinement. Refinement of *F*^2^ against ALL reflections. The weighted *R*-factor *wR* and goodness of fit *S* are based on *F*^2^, conventional *R*-factors *R* are based on *F*, with *F* set to zero for negative *F*^2^. The threshold expression of *F*^2^ > σ(*F*^2^) is used only for calculating *R*-factors(gt) *etc*. and is not relevant to the choice of reflections for refinement. *R*-factors based on *F*^2^ are statistically about twice as large as those based on *F*, and *R*- factors based on ALL data will be even larger.

### Fractional atomic coordinates and isotropic or equivalent isotropic displacement parameters (Å^2^)

**Table:**

	*x*	*y*	*z*	*U*_iso_*/*U*_eq_	
In1	−0.3333	0.3333	−0.5985	0.01029 (5)	
P1	−0.6667	−0.3333	−0.47231 (7)	0.00954 (13)	
P2	0.0000	0.0000	−0.65957 (6)	0.00968 (14)	
O1	−0.15240 (16)	0.15240 (16)	−0.69716 (14)	0.0201 (3)	
O2	−0.5126 (2)	−0.0252 (4)	−0.5013 (2)	0.0276 (4)	
N1	−0.6667	−0.3333	−0.8024 (3)	0.0222 (7)	
HP1	−0.6667	−0.3333	−0.378 (9)	0.027*	
HP2	0.0000	0.0000	−0.553 (6)	0.027*	
HN1	−0.5809 (18)	−0.162 (4)	−0.782 (3)	0.027*	
HN2	−0.6667	−0.3333	−0.8705 (8)	0.027*	

### Atomic displacement parameters (Å^2^)

**Table:**

	*U*^11^	*U*^22^	*U*^33^	*U*^12^	*U*^13^	*U*^23^
In1	0.00741 (6)	0.00741 (6)	0.01606 (8)	0.00371 (3)	0.000	0.000
P1	0.00840 (19)	0.00840 (19)	0.0118 (3)	0.00420 (9)	0.000	0.000
P2	0.00826 (19)	0.00826 (19)	0.0125 (4)	0.00413 (9)	0.000	0.000
O1	0.0275 (7)	0.0275 (7)	0.0188 (6)	0.0240 (8)	−0.0001 (2)	0.0001 (2)
O2	0.0282 (7)	0.0120 (7)	0.0374 (9)	0.0060 (4)	0.0053 (4)	0.0106 (7)
N1	0.0232 (10)	0.0232 (10)	0.0203 (16)	0.0116 (5)	0.000	0.000

### Geometric parameters (Å, º)

**Table:**

In1—O2^i^	2.1226 (19)	P1—O2^iii^	1.5082 (18)
In1—O2	2.1226 (19)	P1—O2	1.5082 (18)
In1—O2^ii^	2.1226 (19)	P1—O2^iv^	1.5082 (18)
In1—O1	2.1461 (17)	P2—O1^v^	1.5255 (16)
In1—O1^ii^	2.1461 (17)	P2—O1^vi^	1.5255 (16)
In1—O1^i^	2.1461 (17)	P2—O1	1.5255 (15)
			
O2^i^—In1—O2	87.75 (10)	O2^ii^—In1—O1^i^	92.35 (6)
O2^i^—In1—O2^ii^	87.75 (10)	O1—In1—O1^i^	87.55 (7)
O2—In1—O2^ii^	87.75 (10)	O1^ii^—In1—O1^i^	87.55 (7)
O2^i^—In1—O1	92.35 (6)	O2^iii^—P1—O2	113.89 (9)
O2—In1—O1	92.35 (6)	O2^iii^—P1—O2^iv^	113.89 (9)
O2^ii^—In1—O1	179.86 (9)	O2—P1—O2^iv^	113.89 (9)
O2^i^—In1—O1^ii^	179.86 (9)	O1^v^—P2—O1^vi^	110.12 (7)
O2—In1—O1^ii^	92.35 (6)	O1^v^—P2—O1	110.12 (7)
O2^ii^—In1—O1^ii^	92.35 (6)	O1^vi^—P2—O1	110.12 (7)
O1—In1—O1^ii^	87.55 (7)	P2—O1—In1	124.21 (11)
O2^i^—In1—O1^i^	92.35 (6)	P1—O2—In1	157.74 (17)
O2—In1—O1^i^	179.86 (10)		

Symmetry codes: (i) −*y*, *x*−*y*+1, *z*; (ii) −*x*+*y*−1, −*x*, *z*; (iii) −*y*−1, *x*−*y*, *z*; (iv) −*x*+*y*−1, −*x*−1, *z*; (v) −*y*, *x*−*y*, *z*; (vi) −*x*+*y*, −*x*, *z*.

### Hydrogen-bond geometry (Å, º)

**Table:**

*D*—H···*A*	*D*—H	H···*A*	*D*···*A*	*D*—H···*A*
N1—H*N*1···O1	0.86 (2)	2.38 (2)	3.066 (2)	138 (2)
N1—H*N*1···O1^ii^	0.86 (2)	2.38 (2)	3.066 (2)	138 (2)
N1—H*N*2···O2^vii^	0.89 (1)	2.41 (1)	3.109 (4)	135 (1)
N1—H*N*2···O2^viii^	0.89 (1)	2.41 (1)	3.109 (4)	135 (1)
N1—H*N*2···O2^ix^	0.89 (1)	2.41 (1)	3.109 (4)	135 (1)
N1—H*N*2···O2^x^	0.89 (1)	2.41 (1)	3.109 (4)	135 (1)

Symmetry codes: (ii) −*x*+*y*−1, −*x*, *z*; (vii) *x*−*y*, *x*, *z*−1/2; (viii) −*x*−1, −*y*, *z*−1/2; (ix) *y*−1, −*x*+*y*−1, *z*−1/2; (x) *y*−1, *x*, *z*−1/2.
